# Boron-substituted 1,3-dienes and heterodienes as key elements in multicomponent processes

**DOI:** 10.3762/bjoc.10.19

**Published:** 2014-01-22

**Authors:** Ludovic Eberlin, Fabien Tripoteau, François Carreaux, Andrew Whiting, Bertrand Carboni

**Affiliations:** 1 Institut des Sciences Chimiques de Rennes, UMR 6226 CNRS-Université de Rennes 1, 263, Avenue du Général Leclerc, Campus de Beaulieu, Bâtiment 10A, 35042 Rennes Cedex, France; 2 Oméga Cat System, 11, allée de Beaulieu, CS 50837, 35708 Rennes Cedex 7, France; 3 Centre for Sustainable Chemical Processes, Department of Chemistry, Durham University, South Road, Durham DH1 3LE, U.K.

**Keywords:** allylboration, boron compounds, Diels–Alder, 1,3-dienes, multicomponent reactions, Petasis borono–Mannich, Suzuki couplings

## Abstract

In the last few years, multicomponent reactions involving boron substituted 1,3-dienes have emerged as important tools in synthetic organic chemistry. The most significant recent results and developments obtained in this area are reported in this review.

## Introduction

Multicomponent reactions involving catalytic or non-catalytic step(s) have become essential tools in the field of synthetic organic chemistry [1–9]. Several of these, which now bear the name of their inventors: Strecker, Hantzsch, Biginelli, Mannich, Passerini or Ugi, have been known and widely used for many years, and the development of new multicomponent processes still receives considerable attention. Indeed, these reactions offer a number of attractive advantages including simple experimental procedures, high convergence, and access to diverse structural and functional systems, often with high levels of atom economy. Boron compounds have long been ignored in this attractive area of research despite their wide range of reactivity [10–11]. In 1993, however, Petasis and co-workers reported a new synthesis of allylamines via stepwise condensation of a secondary amine, paraformaldehyde and (*E*)-styrylboronic acid [12]. This was the first report of this type of transformation, which is now referred as the Petasis borono–Mannich reaction, and was later extended to a wide variety of other aldehydes, such as glyoxylic acid (for example), boronic acids, esters or trifluoroborates and other amine partners [13–15]. Subsequently, other multicomponent reactions involving trialkylborane [16–17], alkenyl- [18–19], aryl- [20–21], allyl- [22], allenyl- [23], and alkynylboronic acids or esters [24–26] have been reported in the literature. Boron-substituted 1,3-dienes and heterodienes have also been successfully used as key elements in such strategies. In this review, we focus on the most significant results and recent contributions obtained in this area [27–28].

## Review

### 1-Boron-substituted 1,3-dienes

**Scheme 1:**
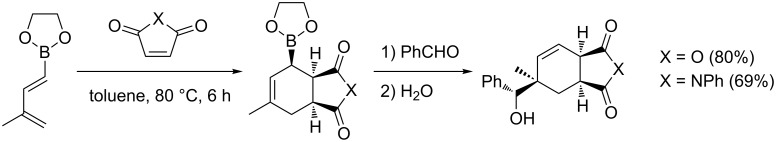
1-Boron-substituted 1,3-diene in a tandem cycloaddition [4 + 2]/allylboration sequence.

The first Diels–Alder reaction involving a 1-boron-substituted 1,3-diene was described in 1972 by Mikhailov and co-workers [29], and it was only fifteen years later that the groups of Vaultier and Hoffmann highlighted the real potential of these compounds in tandem cycloaddition [4 + 2]/allylboration processes [30]. These dienes reacted with activated dienophiles, such as maleic anhydride or maleimides, at relatively high temperatures (80 °C in toluene) to afford exclusively the *cis*-isomers (Scheme 1). The resulting cycloadducts, which contain an allylboronate functionality, then reacted with aldehydes to afford the corresponding homoallylic alcohols with high diastereoselectivity.

**Scheme 2:**
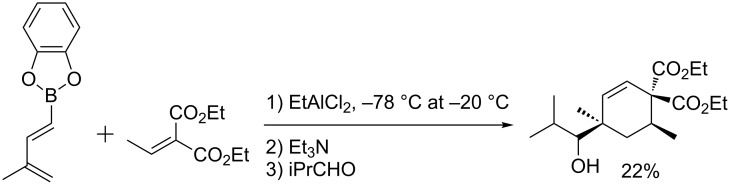
Lewis acid catalyst in the tandem cycloaddition [4 + 2]/allylboration sequence.

In the case of methyl acrylate or acrylonitrile, a mixture of *cis* diastereomers was obtained regioselectively at higher temperature, in a 1:1.6 to 1:1.8 ratio. By contrast, the [4 + 2]-cycloaddition proved to be completely regioselective when performed on the catechol derivative in the absence of solvent [31]. The use of a stoichiometric amount of EtAlCl_2_ as Lewis acid catalyst allowed a lowering of the reaction temperature, a shortening of the reaction time and good stereocontrol (Scheme 2) [32].

**Scheme 3:**
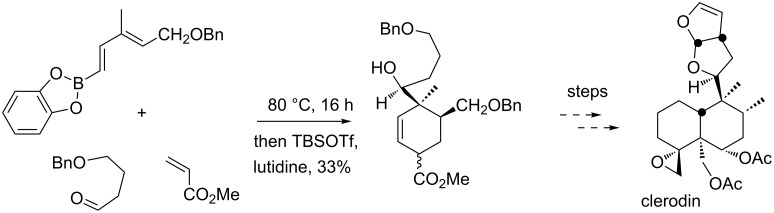
Synthesis of an advanced precursor of clerodin.

Alternatively, the simple heating of a mixture of a 1-bora-1,3-diene, a dienophile and an aldehyde gave direct access to polysubstituted cyclohexenes that were difficult to prepare using the previously reported two step methodology [33]. A concise and efficient synthesis of an advanced precursor of Clerodin, a powerful antifeedant natural product, has been reported using a strategy based on this three-component process (Scheme 3) [34].

**Scheme 4:**
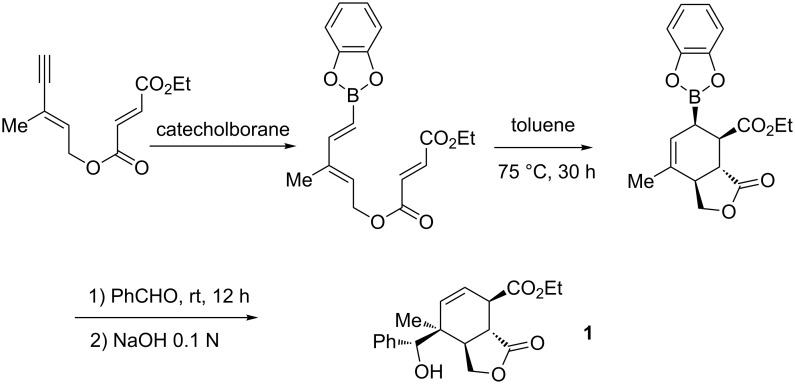
Intramolecular Diels–Alder/allylboration sequence.

Extension of this work to the intramolecular version is depicted in Scheme 4. The bicyclic lactone **1** was obtained stereoselectively from a diene-yne in a one-pot process with control of the relative stereochemistry of the five stereogenic centers [35].

**Scheme 5:**
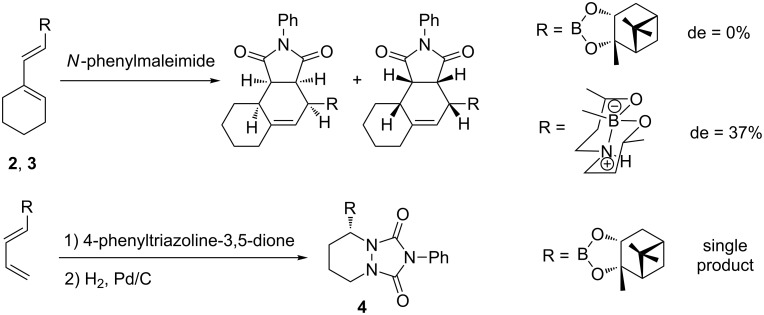
Diastereoselective Diels–Alder reaction with *N*-phenylmaleimide and 4-phenyltriazoline-3,5-dione.

Concerning the access to enantioenriched compounds by using chiral boron substituents, no diastereoselectivity was observed with the (+)-pinanediol ester **2** and *N*-phenylmaleimide [36]. 1,3-Dienyldioxazaborecane **3**, derived from a chiral aminodiol of *C*_2_ symmetry, underwent a faster cycloaddition, as already observed for similar tetracoordinated boron species [37–38], but giving only a modest 2.2:1 ratio of diastereoisomers. By contrast, the cycloadduct **4** was obtained as a single product with excellent stereoselectivity in 84% yield, however, this could probably be attributed to the special structure of the dienophile used (Scheme 5) [39].

**Scheme 6:**
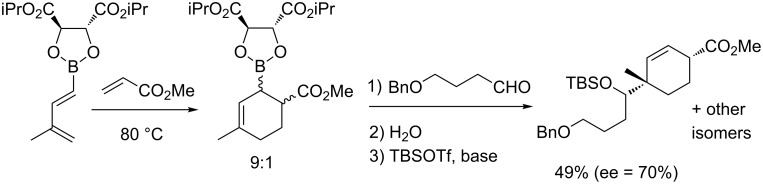
Asymmetric synthesis of a α-hydroxyalkylcyclohexane.

Finally, interesting results were reported with methyl acrylate and dienes derived from tartrate esters (9/1 de, 70% ee for the major isomer) (Scheme 6) [40].

**Scheme 7:**
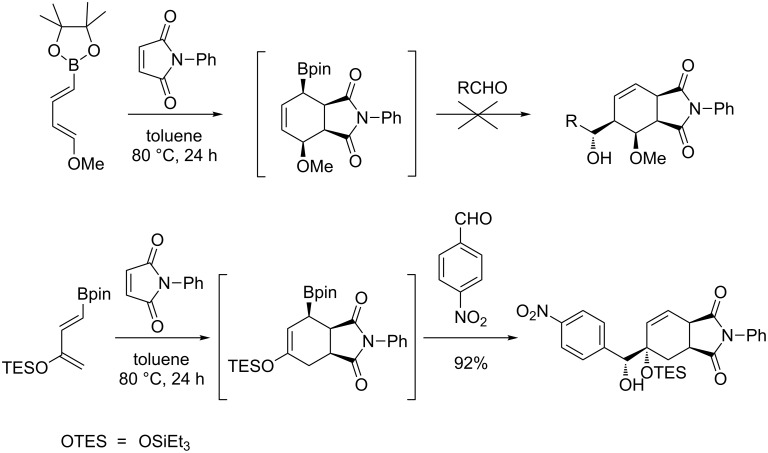
Tandem [4 + 2]-cycloaddition/allylboration of 3-silyloxy- and 4-alkoxy-dienyl boronates.

In 2003, Hall and co-workers reported the application of electron-rich dienylboronates in one-pot tandem Diels–Alder/allylboration reactions [41]. In the case of the 4-methoxy-substituted diene, if the first step occurred at 80 °C in toluene, it was impossible to obtain the allylation products, even by heating at higher temperature or by activation with EtAlCl_2_. By contrast, with the 3-OTES derivative, bicyclic, three-component adducts were isolated in good yields up to 92%. A single diastereomer was detected with maleimides; the diastereoselectivity being lower with methyl acrylate and vinyl oxazolidinone (Scheme 7).

**Scheme 8:**
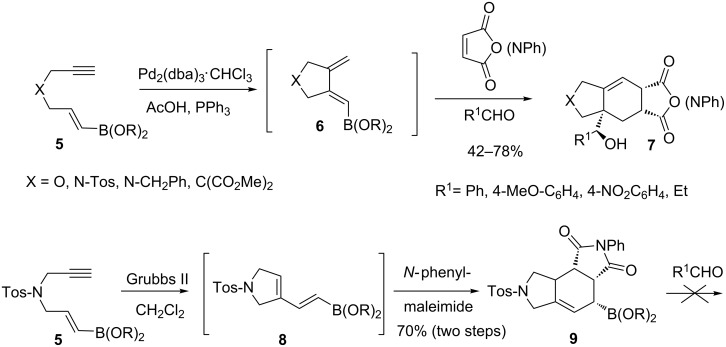
Metal-mediated cycloisomerization/Diels–Alder reaction/allylboration sequence.

A one-pot, palladium-mediated cycloisomerization of ene-ynes **5** was applied to the synthesis of the boronated dienes **6**, which were not isolated, but directly used in a one-pot Diels–Alder reaction/allylboration sequence. This efficiently generated, in high yields, tricyclic structures **7** with control of four stereogenic centers created (Scheme 8). In the presence of Grubbs II catalyst, a skeletal isomer **8** was produced from **5**. If the [4 + 2]-cycloadduct **9** was obtained with *N*-phenylmaleimide, it failed to give homoallylic alcohols, probably due to steric hindrance [42].

**Scheme 9:**
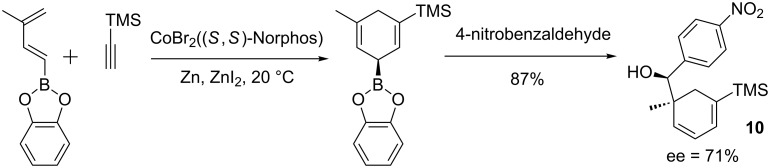
Cobalt-catalyzed Diels–Alder/allylboration sequence.

An elegant three-component process was developed by Hilt and co-workers using a cobalt-catalyzed Diels–Alder reaction as the key step in a one-pot, cycloaddition/allylboration sequence [43–44]. With (*S,S*)-Norphos as chiral ligand, the desired alcohol **10** was isolated in 87% yield and 71% ee (Scheme 9).

**Scheme 10:**
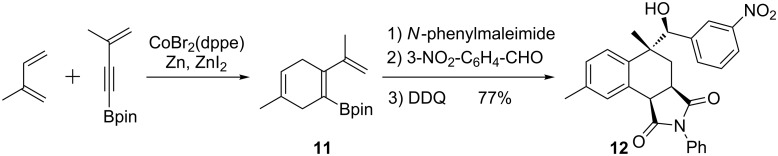
A two-step reaction sequence for the synthesis of tetrahydronaphthalenes **12**.

In a related process, the same group reported a two-step reaction cascade interconnecting four components to afford dihydroaromatic compounds **11** with a high degree of diastereoselectivity and good yields. After cycloaddition and allylboration, the resulting product was oxidized to afford the corresponding tetrahydronaphthalenes **12** (Scheme 10) [45].

**Scheme 11:**
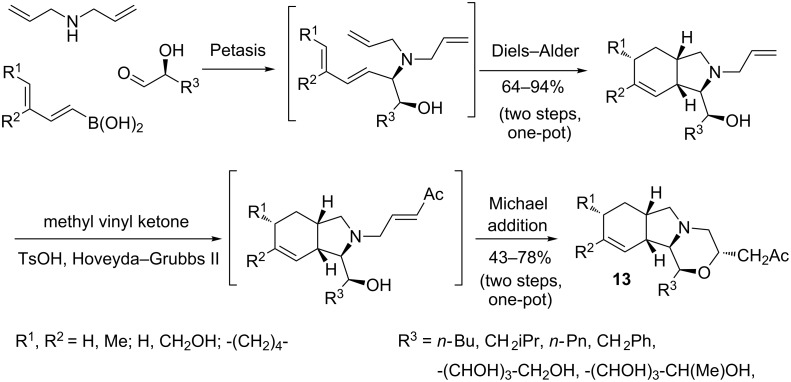
Tandem sequence based on the Petasis borono–Mannich reaction as first key step.

Most of the tandem sequences, in which boron-substituted 1,3-dienes were involved, were based on a first Diels–Alder cycloaddition, as shown above. However, a recent report of Norsikian, Beau and co-workers described a novel sequence of tandem transformations which combined the Petasis reaction, intramolecular [4 + 2]-cycloaddition, cross metathesis and Michael reaction. This process gave access to new polycyclic heterocyclic scaffolds **13** with good yields and complete stereocontrol (Scheme 11) [46].

### 2-Boron-substituted 1,3-dienes

**Scheme 12:**
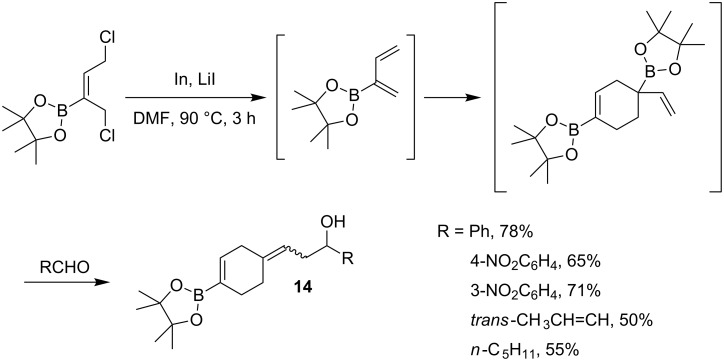
One-pot tandem dimerization/allylboration reaction of 1,3-diene-2-boronate.

In contrast to 1,3-dienes functionalized with a boron atom in position 1, only a few studies have been reported on the related dienes substituted in position 2. These compounds are often difficult to synthesize and have, at least for the less-substituted derivatives, a strong tendency to undergo Diels–Alder dimerization even at room temperature [47–48]. This process was carefully investigated by Carreaux, Cossio and co-workers with regard to theoretical and experimental aspects [49]. When the dechlorination was carried out in the presence of an aldehyde, the dimer was immediately converted to the expected corresponding homoallylic alcohols **14** in moderate to good yields as mixtures of *E*/*Z* isomers (Scheme 12).

**Scheme 13:**
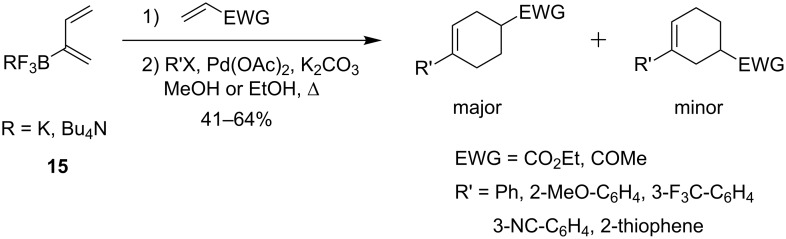
Tandem Diels–Alder/cross-coupling reactions of trifluoroborates **15**.

In 2005, Welker and co-workers started a series of studies on the synthesis of 2-boron-substituted 1,3-dienes and their reactivity in tandem reactions, concentrating mainly upon [4 + 2]-cycloadditions followed by cross-coupling reactions. Potassium and tetra-*n*-butylammonium buta-1,3-dienyl-2-trifluoroborates **15** were synthesized in good yields from chloroprene and showed no propensity to dimerize [50–51]. Exploration of the reactivity of these dienyl trifluoroborates began with Diels–Alder reactions with ethyl acrylate, methyl vinyl ketone and *N*-phenylmaleimide. The boron-containing cycloadducts, isolated with high yields, were subsequently cross-coupled using palladium catalysis. A one-pot sequence was also developed, first heating the boron diene with the dienophile, then adding an aryl halide, Pd(OAc)_2_ (5 mol %), K_2_CO_3_ (3 equiv) and finally refluxing the mixture in EtOH or MeOH for 5 h (Scheme 13). Reactions with various aryl halides, substituted by electron-donating or -withdrawing groups and heteroaromatic halides occurred in moderate to good yields (41% to 64% over two steps) with a preference for the *para-* over *meta*-regioisomers (2.3:1 to 5.7:1 ratio).

**Scheme 14:**
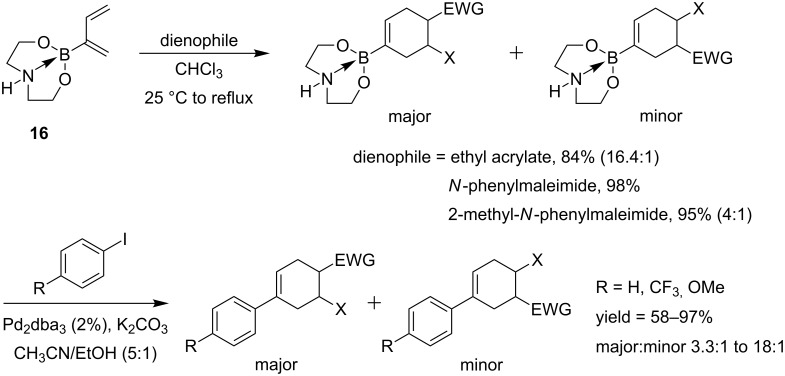
Diels–Alder/cross-coupling reactions of **16**.

Using the same methodology, the preparation and reactivity in tandem Diels–Alder/palladium cross-coupling sequences of a 2-diethanolaminoboron-substituted 1,3-diene **16** were investigated [52]. This diene has proved to be significantly more reactive and more regioselective in [4 + 2]-cycloadditions compared to the corresponding trifluoroborate. The cycloadducts were then efficiently cross-coupled to iodobenzene, 4-trifluoromethyl-1-iodobenzene and 4-iodoanisole. The regioselectivities observed in the initial Diels–Alder reactions were maintained after cross-coupling (Scheme 14).

More recently, new 2-boron-substituted 1,3 dienes containing diol and triol ligands were prepared under basic conditions that prevent the dimerization by-product formation. These four-coordinate boron complexes participated in the same tandem Diels–Alder/Suzuki cross-coupling sequence, which appeared to be palladium- catalysed. The final cycloadducts were obtained in good yields (63% to 83%) [53].

**Scheme 15:**
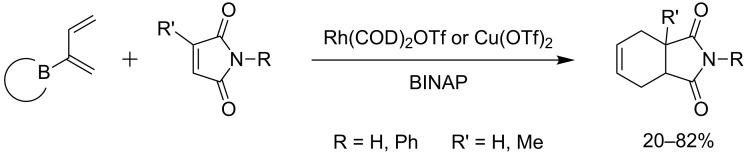
Metal catalyzed tandem Diels–Alder/hydrolysis reactions.

Finally, Welker and co-workers described metal-catalysed tandem Diels–Alder/hydrolysis reactions of 2-boron-substituted 1,3-dienes [54–55]. Boron-dienes containing various ligands reacted with maleimides in the presence of rhodium and copper catalysts using BINAP as ligand (Scheme 15). NMR analysis of the crude product showed traces of the boron cycloadduct, highlighting that this mechanism proceeds, first with a Lewis-acid catalysed [4 + 2]-cycloaddition, and then by Lewis acid-assisted C–B bond protonation.

**Scheme 16:**
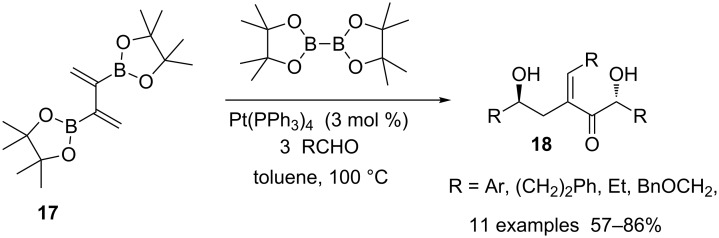
Synthesis of *anti*-1,5-diols **18** by triple aldehyde addition.

In a different approach which exploited another aspect of the reactivity of boron-substituted dienes, 2,3-bis[(pinacolato)boryl]-1,3-diene **17**, synthesized by treatment of 1,1-[bis(pinacolato)boryl]alkenes with excess of 1-bromo-1-lithioethene [56–57], were engaged in a triple aldehyde addition. 1,5-*anti*-Diols **18** were produced via successive Pt-catalyzed 1,4-diboration, allylboration reactions and finally elimination of boryl and boroxy groups. Four C–B bonds were converted into two C–C and one C=C bonds with total stereocontrol in each step (Scheme 16) [58].

### 1-Boron-substituted 1,3-heterodienes

#### 1-Oxo-4-borono-1,3-dienes

**Scheme 17:**
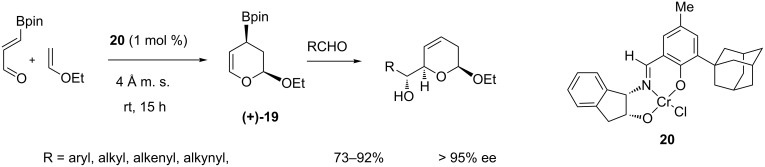
Catalytic enantioselective three-component hetero-[4 + 2]-cycloaddition/allylboration sequence.

In 2003, first examples of a novel diastereoselective routes to α-hydroxyalkyldihydropyrans were reported; a substructure frequently encountered in the core of a wide range of natural products [59–61]. As in the carbocylic variant, the intermediate cyclic allylboronate (+)-**19**, prepared from 3-boronoacrolein, was the key element of a sequential Diels–Alder/allylation. In this case, the catalytic asymmetric version was carried out efficiently in the presence of the Jacobsen’s tridentate chromium(III) complex **20** catalyst with high diastereo- and enantioselectivity (Scheme 17).

**Scheme 18:**
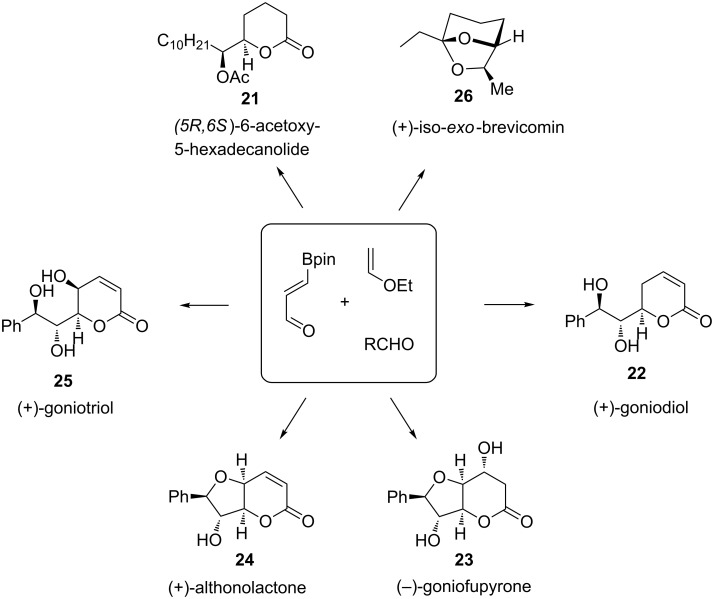
Synthesis of natural products using the catalytic enantioselective HDA/allylboration sequence.

The first application of this strategy in the synthesis of natural products and analogues concerned (*5R,6S*)-6-acetoxy-5-hexadecanolide **21**, the oviposition attractant pheromone of a mosquito capable of transmitting the West Nile virus [59]. Thereafter, several members of the natural styryllactone family **21**–**25**, displaying cytotoxic and antitumor activities, have been also prepared according to this methodology [62–64]. The combination of the catalytic hetero-Diels–Alder/allyboration sequence with a ruthenium-catalyzed isomerization gave access to the 6,8-dioxabicyclo[3.2.1]octane skeleton of (+)-iso-*exo*-brevicomin (**26**, Scheme 18) [65].

**Scheme 19:**
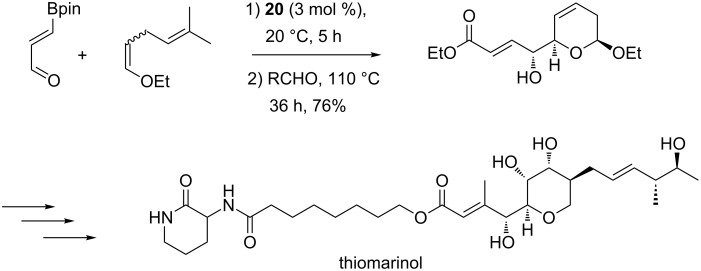
Total synthesis of a thiomarinol derivative.

When a *Z*/*E* mixture of 2-substituted enol ethers was used as dienophile, only cycloadducts resulting from a selective reaction of the *Z*-isomer were present in the final product that prevented the tedious preparation of an isomerically pure starting material. While the second allylboration step required conditions more severe than those employed in the case of ethyl vinyl ether, this approach was successfully applied in the key steps of the total synthesis of a member of the thiomarinol class of marine antibiotics (Scheme 19) [66–67].

**Scheme 20:**
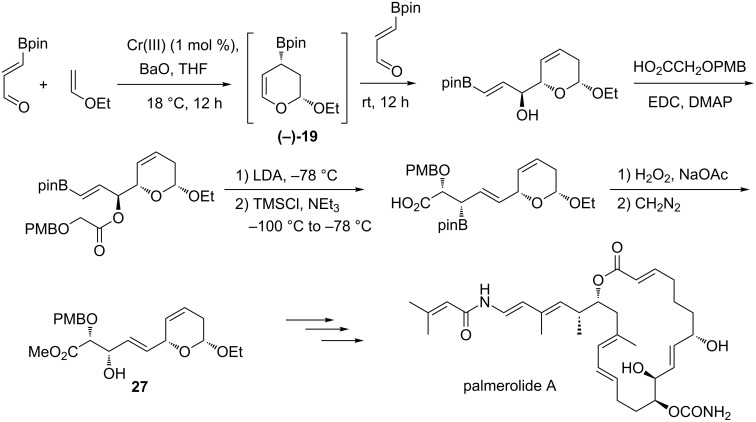
Synthesis of an advanced intermediate **27** for the east fragment of palmerolide A.

More recently, Hall and co-workers described the total synthesis of a complex 20-membered marine macrolide, palmerolide A, a potent and unusually selective antitumor agent [68]. In this case, the cyclic allylboronate (−)-**19** prepared from the [4 + 2]-cycloaddition reacted with the starting 3-boronoacrolein which then played the role of the aldehyde partner. The hydroxy group of the resulting homoallylic alcohol was then engaged in a Claisen–Ireland rearrangement to afford an advanced intermediate **27** for the completion of the total synthesis (Scheme 20).

#### 1-Aza-4-borono-1,3-dienes

**Scheme 21:**
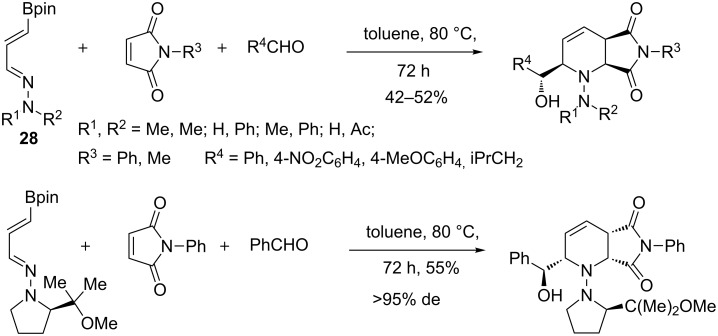
Bicyclic piperidines from tandem aza-[4 + 2]-cycloaddition/allylboration.

A few years before the description of the tandem process concerning the 3-boronoacrolein, Hall and co-workers realized a multicomponent reaction involving 1-aza-4-borono-1,3-dienes **28** as key starting materials for the preparation of α-hydroxyalkylpiperidines. These scaffolds are present in several natural products, particularly in the alkaloid family of palustrine. The tandem process started with the hetero-[4 + 2]-cycloaddition of an hydrazonodiene with maleimides, as electron-poor dienophiles, followed by an allylboration (Scheme 21) [69]. This sequence proceeded with high stereocontrol, as already observed with the carbon and oxygen analogues. In addition, the absolute stereochemistry of the four new created stereogenic centers was controlled by using a chiral auxiliary (>95% de in the case of an L-proline-derived diene).

**Scheme 22:**
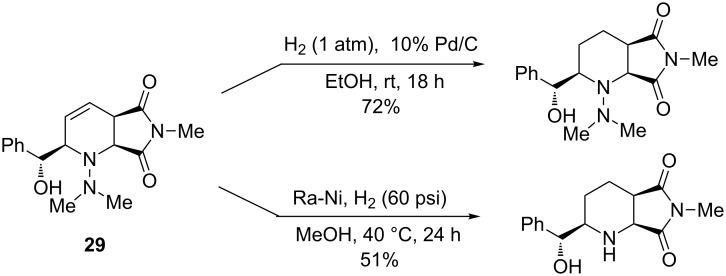
Hydrogenolysis reactions of hydrazinopiperidines.

Diversification on the different units, diene, dienophile and aldehyde, has been described. Concerning the maleimide material, substituent R^3^ did not exert any significant effect on the process. Other dienophiles have also been tested (acrylates, maleic anhydride) giving no products or unsatisfactory results. A large range of aldehydes, as aliphatic, electron-rich or electron-poor benzaldehyde, could be used. Only very hindered aldehydes did not afford any product and both mono- and disubstituted arylhydrazines have proved to give the best yields, probably due to the higher reactivity of the corresponding hydrazones. Furthermore, the double bond of **29** was selectively hydrogenated under palladium on charcoal, while hydrogenolysis of the hydrazine moiety occurred in the presence of Raney nickel (Scheme 22).

Following these results obtained in the liquid phase, Hall and co-workers also examined the suitability of a solid-phase strategy. Finally, due to problems of purity encountered with an *N*-arylmaleidobenzoic acid-functionalized resin [70], or availability of the supported aldehyde partner, a four-component variant of this chemistry was developed in solution phase. The pre-formation of the azabutadiene component was not necessary and this gave access to a library of 944 polysubstituted piperidines with sufficient purity suitable for biological screening after purification by HPLC with mass-based fraction collection [71].

**Scheme 23:**
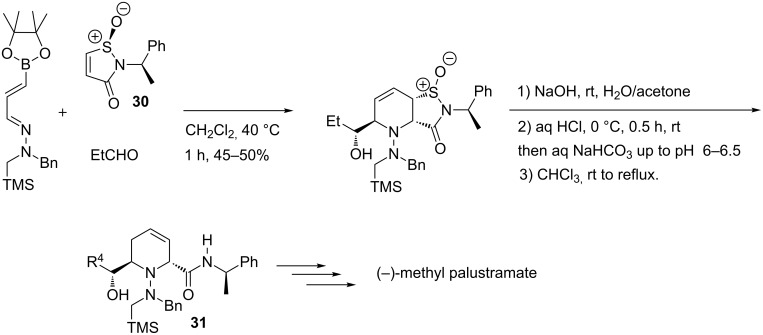
Tandem aza-[4 + 2]-cycloaddition/allylboration/retrosulfinyl-ene sequence.

The flexibility and usefulness of this chemistry was also demonstrated in target-oriented synthesis with the synthesis of (−)-methyl palustramate and other 2,6-disubstituted piperidines [72–73]. A chiral sulfinimide **30** was used as dienophile and a sequential three component aza-[4 + 2]-cycloaddition/allylboration/retro-sulfinyl-ene sequence was performed to afford 1,2,5,6-tetrahydropyridine **31** with high regio- and diastereoselectivity (Scheme 23).

### Boron-substituted heterodendralene

**Scheme 24:**
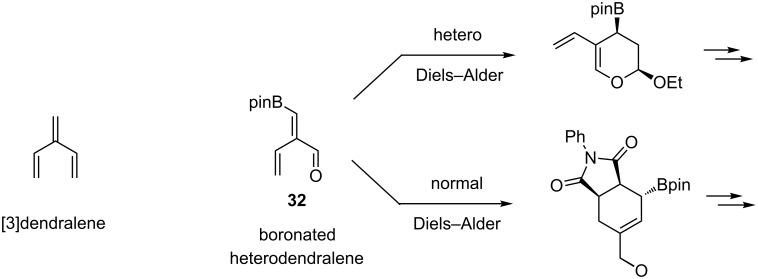
Boronated heterodendralene **32** in [4 + 2]-cycloadditions.

On the basis of these precedents, boronated 2-vinyl-α,β-unsaturated aldehydes **32** were designed to fully exploit the synthetic potential of these classes of organoboranes. These compounds, named boronated [3]-1-heterodendralenes by analogy with the corresponding carbotrienes [74], have been used to synthesize polycyclic heterocycles with control of multiple stereocenters [75]. Based on the intrinsic reactivity of each 1,3-dienyl system, sequential hetero-Diels–Alder/Diels–Alder reactions (or vice versa if the order of introduction of the reagents was inversed) were carried out chemoselectively. The allylboronic ester moiety, generated in the first cycloaddition step or after the two consecutive [4 + 2]-cycloadditons, can further be engaged in an allylation reaction that significantly increased the structural diversity of the final products (Scheme 24).

**Scheme 25:**
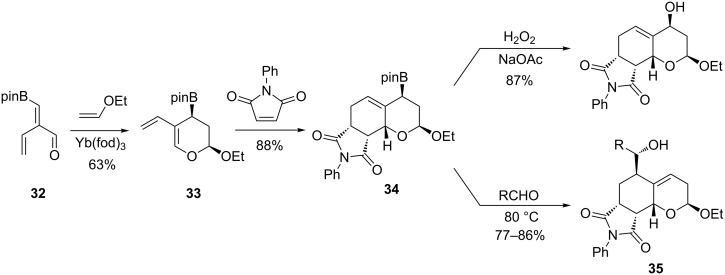
Synthesis of tricyclic imides derivatives.

As 3-boronoacrolein esters which have been used in metal-catalyzed inverse electron demand [4 + 2]-cycloadditions, **32** reacted with ethyl vinyl ether in the presence of Yb(fod)_3_ to afford 2-alkoxy-3,4-dihydro-5-vinyl-2*H*-pyran **33**. In the presence of electron-poor dienophiles, as *N*-phenylmaleimide, maleic anhydride, activated azo compounds or naphthoquinone, **33** underwent normal Diels–Alder reactions thus giving the corresponding cycloadducts **34** as single diastereomers (Scheme 25). No formation of products resulting from a first cycloaddition of the 1,3-butadienyl moiety was observed when these reactions were conducted in a tandem one-pot process. Various transformations were further carried out as oxidation with hydrogen peroxide or addition to aldehydes that gave access to the homoallylic alcohols **35** in 77–86% yields.

**Scheme 26:**
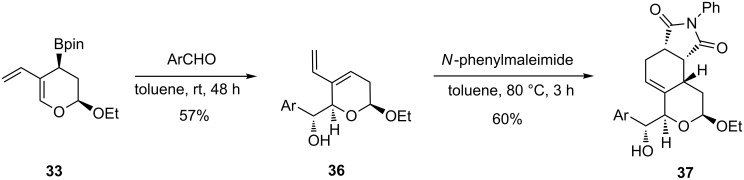
Synthesis of **37** via a HDA/allylboration/DA sequence.

Alternatively, the presence of an allylic boronic ester group in the cycloadduct **33** was exploited in carrying out first the addition to 4-nitrobenzaldehyde to afford **36**. Further normal-electron demand [4 + 2]-cycloaddition step with *N*-phenylmaleimide furnished the single tricyclic compound **37** (Scheme 26).

**Scheme 27:**
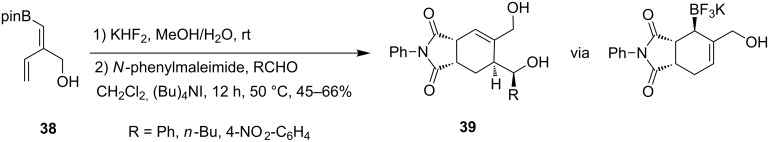
Diels–Alder/allylboration sequence.

The initial boronic ester group of **38**, the direct precursor of **32**, can be also converted into a trifluoroborate substituent by treatment with KHF_2_ in MeOH/H_2_O to increase the reactivity of the dienyl moiety towards electron-poor dienophiles. It was engaged in a one pot Diels–Alder cycloaddition with *N*-phenylmaleimide in the presence of various aldehydes to afford diols **39** as major diastereisomers (>95%) and in good overall yields (four steps) (Scheme 27).

## Conclusion

Despite the synthetic potential of the boron-substituted 1,3-dienes and heterodienes presented herein in creating molecular complexity, the field of multicomponent reactions involving these versatile building blocks remains insufficiently explored. If Diels–Alder cycloadditions have been mainly employed as key steps in most of the reported processes, numerous other reactions can be envisaged. Further developments in this area will certainly provide important improvements with regards to the scope of reagents, access to new structural scaffolds with control of the regio-, diastereo- and enantioselectivity and the efficiency of these multistep sequences.
